# Viruses and miRNAs: More Friends than Foes

**DOI:** 10.3389/fmicb.2017.00824

**Published:** 2017-05-15

**Authors:** Patrice Bruscella, Silvia Bottini, Camille Baudesson, Jean-Michel Pawlotsky, Cyrille Feray, Michele Trabucchi

**Affiliations:** ^1^INSERM U955, Team “Pathophysiology and Therapy of Chronic Viral Hepatitis”, Université Paris-EstCréteil, France; ^2^INSERM, C3M, Université Côte d’AzurNice, France

**Keywords:** microRNAs, viral microRNAs, virus life cycle, gene expression, clinical perspectives

## Abstract

There is evidence that eukaryotic miRNAs (hereafter called host miRNAs) play a role in the replication and propagation of viruses. Expression or targeting of host miRNAs can be involved in cellular antiviral responses. Most times host miRNAs play a role in viral life-cycles and promote infection through complex regulatory pathways. miRNAs can also be encoded by a viral genome and be expressed in the host cell. Viral miRNAs can share common sequences with host miRNAs or have totally different sequences. They can regulate a variety of biological processes involved in viral infection, including apoptosis, evasion of the immune response, or modulation of viral life-cycle phases. Overall, virus/miRNA pathway interaction is defined by a plethora of complex mechanisms, though not yet fully understood. This article review summarizes recent advances and novel biological concepts related to the understanding of miRNA expression, control and function during viral infections. The article also discusses potential therapeutic applications of this particular host–pathogen interaction.

## Background

To date, different classes of regulatory small-RNAs have been identified which differ in their biogenesis, length, and tissue distribution (Ghildiyal and Zamore, 2009; Kim et al., 2009). Among the small RNAs are microRNAs (miRNAs). miRNAs are involved in the control of a broad range of cellular activities, such as development, immune function, and cell death (Sayed and Abdellatif, 2011). Genes can encode single miRNAs or clusters of two or more miRNAs, which are processed from common primary transcripts, called pri-miRNAs (Lee et al., 2004). The pri-miRNAs are processed by the nuclear RNAse III endonuclease DROSHA to generate intermediate precursors of about 70 nucleotides (nt) in length, named pre-miRNAs. The pre-miRNAs are further processed by the cytoplasmic RNAse III endonuclease Dicer to generate mature miRNAs of about 22 nt in length. Proteomic analyses revealed the presence of several RNA-binding proteins that regulate miRNA biogenesis (Gregory et al., 2006; Guil and Caceres, 2007; Trabucchi et al., 2009; Suzuki et al., 2011) in both physiological and pathological contexts (Frezzetti et al., 2011; Abdelmohsen et al., 2012; Repetto et al., 2012; Emde et al., 2015; Hata and Lieberman, 2015).

Mature miRNAs are loaded into Argonaute proteins (mainly AGO2) to form the miRNA-induced silencing complex (miRISC) (Ha and Kim, 2014). One strand of the mature miRNA (the guide strand) targets mRNAs to promote degradation or translational blockade in the Processing-Bodies (Kulkarni et al., 2010). Canonically, miRNAs target the 3′ untranslated region (3′-UTR) or the coding sequence (CDS) of mRNAs by base pairing with nucleotides 2–7 of the miRNA 5′ end, which is called the seed-sequence (Lewis et al., 2003, 2005; Brennecke et al., 2005; Friedman et al., 2009; Pasquinelli, 2012). Some studies claim, however, that about 60% of miRNA binding activity is non-canonical, and involves portions of miRNA sequences located outside of the seed and/or with seed-like motifs including mismatches or bulges (Chi et al., 2012; Helwak et al., 2013).

MicroRNAs were shown to play a role in the replication and propagation of viruses, including cellular antiviral responses and/or in the promotion of viral infections through complex regulatory pathways. It was also shown that miRNAs encoded by viral genomes can be expressed in host cells and participate in the lifecycle and in the cellular consequences of infection. This article review focuses on recent advances in the understanding of miRNA expression and function during viral infections, aiming at providing insights into their role in the pathogenesis of these infections. The article also discusses future directions for research and clinical use of miRNAs as antiviral agents.

## Viruses Induce Host miRNAs and Regulate their Turnover and Function

In early phases of viral infections, innate sensors of host cells detect viral products and initiate signal cascades involved in the antiviral responses. Antiviral miRNAs are components of this response. Some viruses have the capacity of manipulating host miRNAs into escaping an antiviral response or to promote viral infection.

### Viral Infections Can Induce the Transcription of Host miRNAs Involved in the Antiviral Response

To initiate and maintain an antiviral innate response while the more specific adaptive response takes place, cells have developed a microbial pathogen recognition system based on pathogen recognition receptors (PRRs). PRRs, which include Toll-like receptors (TLRs), retinoic acid-inducible gene I-like receptors and Nod-like receptors, recognize a broad spectrum of motifs common to viral pathogens and this recognition activates a downstream antiviral cascade that includes miRNAs (Li and Shi, 2013). For instance, in hepatitis C virus (HCV)-infected hepatocytes, type I interferon (IFNα/β) production caused by endosomal TLR activation rapidly modulates the expression of numerous host miRNAs, including miR-196, miR-296, miR-351, miR-431 and miR-448, that target the HCV RNA genome with the aim to inhibit viral replication (Pedersen et al., 2007).

**FIGURE 1:**
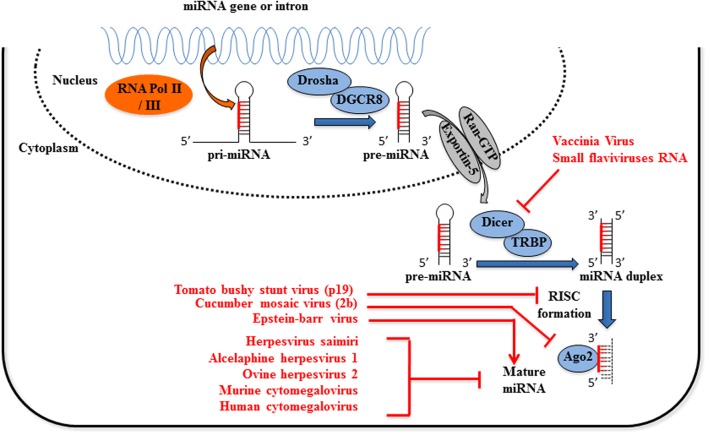
**Viruses interfere with host miRNA biogenesis**. Primary miRNAs (pri-miRNAs) processing, first to precursor miRNAs (pre-miRNAs) and then to mature miRNAs, involves two ordered endonucleolytic cleavages. Following transcription by RNA polymerase II/III, the multiprotein complex containing Drosha processes the pri-miRNA into a ∼70 nt hairpin pre-miRNA. Through the interaction with exportin-5 and Ran-GTP, the pre-miRNA is transported into the cytoplasm where it undergoes a second round of processing catalyzed by Dicer. One strand of the resulting small RNA duplex, the mature miRNA, is loaded into the RNA induced silencing complex (RISC) which post-transcriptionally regulates the expression of target genes. Expression of viral proteins termed viral suppressor of RNA silencing inhibits the loading of miRNAs into the RISC complex (tomato bushy stunt virus p19 protein) or Ago2 activity (cucumber virus 2b protein). Multiple processes mediated by Epstein–Barr virus are responsible for miR-155 upregulation, among which chromatin remodeling, cell signaling regulation and transcription factor activation. Flaviviruses sfRNA and Vaccinia virus inhibit or reduce the expression of Dicer activity, respectively. Finally, several Herpesviruses encode viral sequences complementary to mature miRNAs miR-17 and miR-27, leading to their degradation or the inhibition of the miRNA-induced regulation of mRNA targets.

Virus-dependent activation of PRRs can also induce the expression of miRNAs able to promote innate immunity through a positive feedback regulatory loop. In an infection by the vesicular stomatitis virus (VSV), miR-155 and miR-223 expression is induced in macrophages through a RIG-I/JNK/NF-κB-dependent pathway. MiR-155 expression is also induced in an infection with Epstein–Barr virus (EBV) in B-cell lymphomas (**Figure 1**), by a mechanism involving the Activator protein 1 (AP1) transcription factor and DNA hypomethylation (Yin et al., 2016).

In turn, both miRNAs promote the type I IFN-mediated antiviral response by suppressing cytokine signaling 1 (SOCS1) (Wang et al., 2010) and FOXO3 (Chen et al., 2016), respectively. Another example of SOCS1 suppression was observed in patient developing dengue hemorrhagic fever (DHF) and this suppression is associated with elevated levels of miR-150 in CD14+ monocytes infected with DENV2 (Chen et al., 2014).

### Viral Infections Can Inhibit the Maturation of Host miRNAs Involved in the Antiviral Response

The role played by miRNAs in antiviral defenses can be counteracted by viruses that deploy specific virulence factors, referred to as viral suppressors of RNA silencing (VSRs), which target several components of the host silencing machinery including small RNA processing, stability and activity *via* AGO effectors (Wu et al., 2010). The 2b protein of the cucumber mosaic virus (CMV) inhibits AGO1 slicer function independently of its dsRNA-binding activity (Feng et al., 2013), while the p19 protein of the tomato bushy stunt virus (TBSV) inhibits miRISC loading activity by binding to small double-stranded (ds)RNAs (Lakatos et al., 2006). These mechanisms promote viral replication.

Flaviviruses, which are single-stranded (ss) positive-RNA viruses, interfere with the miRNA processing machinery through an accumulation of the non-coding (nc) subgenomic flavivirus RNA (sfRNAs) that induces sequestration of the dsRNA binding proteins Dicer and AGO2 (Moon et al., 2015). This viral strategy is put in place by different flaviviruses, including Murray valley encephalitis virus (MVEV), Japanese encephalitis virus (JEV), West nile virus (WNV), Yellow fever virus (YFV), and Dengue virus (DENV) (Schnettler et al., 2012; Bavia et al., 2016).

An indirect regulation of miRNA biogenesis has been observed in HeLa cells infected by vaccinia virus (VACV), a member of the *Poxviridae* family with a linear double-stranded DNA genome. VACV abrogates the expression of Dicer independently of VACV decapping enzymes *via* a mechanism that remains to be clarified (Grinberg et al., 2012) (**Figure 1**).

### Viral Infections Can Accelerate the Degradation of Host miRNAs Involved in the Antiviral Response

Different viruses encode ncRNAs that contain sequences complementary to those of host miRNAs, thereby promoting their degradation. The γ-herpesvirus saimiri (HVS) expresses a small U-rich ncRNA, HSUR 1, which contains a sequence complementarity to miR-27 (Cazalla et al., 2010). Binding with HSUR 1 induces miR-27 degradation and this down-regulation activates T-cells during HVS infection (Guo et al., 2014). Recently, it was demonstrated that this mechanism was dependent of a flexible conformation of the miR-27 binding region on HSUR 1 (Pawlica et al., 2016). This mechanism has also been reported with the Murine cytomegalovirus (MCMV) (Libri et al., 2012; Marcinowski et al., 2012). Two other oncogenic γ-herpesviruses, alcelaphine herpesvirus 1 and ovine herpesvirus 2, encode viral homologes of miR-27 target genes leading to rapid decay of this miRNA (Guo et al., 2014).

Human cytomegalovirus (HCMV) expresses an nc-transcript, called miRNA decay element (miRDE), that contains several binding sites for miR-17 family members, the binding of which causes their degradation. This proviral function leads to an upregulation of viral DNA synthesis and viral production during lytic infection (Lee et al., 2013) (**Figure 1**).

These examples show that viral infections can trigger the expression of antiviral miRNAs, but can also regulate miRNA turnover and function in order to favor their own propagation. The mechanisms by which miRNAs are degraded after a viral infection need to be clarified but could involve 3′-end addition of non-templated nucleotides by tailing enzymes, followed by degradation by exonucleases (Ameres et al., 2010; Marcinowski et al., 2012; McCaskill et al., 2015).

## Host miRNAs Control Viral RNA Production and Turnover

Host miRNAs have been reported to directly target viral RNAs to promote or inhibit the viral lifecycle.

### Host miRNAs Can Directly Block Viral Replication

Elevated levels of miR-296-5p were detected in Enterovirus 71 (EV71)-infected human rhabdomyosarcoma (RD) and SK-N-Sh cells. MiR-296-5p targets both capsid protein VP1 and VP3 coding regions in the viral genome as a response to viral infection (Zheng et al., 2013). Other miRNAs inhibit EV71 replication, such as miR-23b that targets the EV71 VP1 RNA coding region (Ho et al., 2016).

Coxsackievirus B3 (CVB3) is an RNA virus belonging to the *Picornaviridae* family that provokes cardiomyopathies. In HeLa cells, miR-342-5p targets the 2C-coding region of the viral RNA, which results in its degradation (Wang et al., 2012).

Herpes simplex virus type 1 (HSV-1) replicates in epithelial cells and persists in a latent form in sensory neurons. Lytic replication and reactivation from latency depend on the expression of viral Infected Cell Protein 0 (ICP0), which is controlled by the cell-specific miR-138 in neurons (Pan et al., 2014).

Another example is provided by miR-548g-3p that targets the Stem Loop A promoter element of DENV 5′-UTR, inhibiting the recruitment of the viral RNA-dependent RNA polymerase (NS5) to the viral genome and resulting in a blockade of viral replication (Wen et al., 2015).

### Host miRNAs Can Suppress Proviral Host Factors

Host miRNAs can play an antiviral role by targeting host mRNAs that encode proviral proteins. In human EAhy926 cells infected with DENV-2, miR-223 downregulates the microtubule-destabilizing protein stathmin 1 (STMN1), thereby inhibiting viral replication (Wu et al., 2014). MiR-199a-3p inhibits the replication of different viruses, including herpesviruses and alphaviruses, *via* downregulation of ERK/MAPK, oxidative stress and PI3K/AKT pathway activation (Santhakumar et al., 2010). The WNV_KUN_-induced miR-532-5p downregulates SESTD1 (SEC14 and spectrin domains 1) and TAB3 (TGF-beta activated kinase 1/MAP3K7 binding protein 3) mRNAs to block WNV replication (Slonchak et al., 2015).

### Host miRNAs Can Act As Proviral Factors through Direct Interaction with the Viral Genome

**FIGURE 2:**
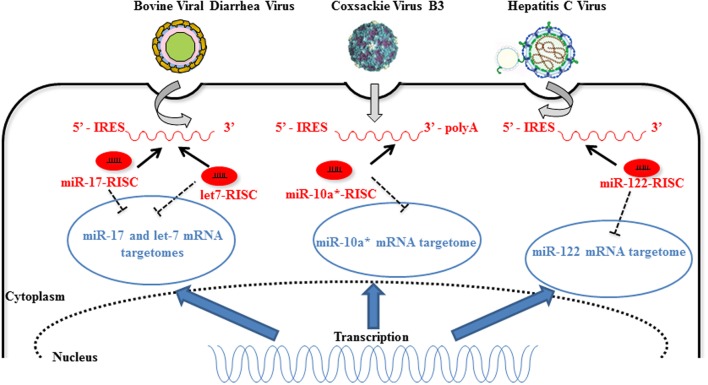
**Host miRNAs directly improve RNA virus replication**. Direct interaction of 3′ end- bovine viral diarrhea virus (BVDV), 3D-coding of region Coxsackie virus B3 (CVB3), and 5′-IRES of hepatitis C virus (HCV) RNAs with host miR-17, let-7, miR-10a-3p, and miR-122, respectively, increases viral replication (dark arrows). The unusual interaction between host miRNA and increasing amounts of viral RNA during replication implies a diminution of the interaction of the host miRNA with its cellular targets (“sponge effect”) (dark dotted inhibition arrows). MiR-10a-3p targets mRNAs implicated in temozolomide resistance (Ujifuku et al., 2010) indicating that miR-10a-3p is not only a passenger miRNA but has a functional role in the cells [image source for CVB3 (Luo et al., 2010) and HCV (Lindenbach and Rice, 2013)].

Competition for miRNA binding has been reported in the case of human diseases and is termed “competitive viral and host RNAs” (cvhRNA) (Li et al., 2014). In infected cells, the interaction between viral RNAs and host miRNAs could be necessary for viral RNA stability, replication, or infection (Jopling et al., 2005). Therefore, viral RNAs harboring common miRNA-binding sites with host mRNA could act as sponges and sequester endogenous miRNAs. *In fine*, the stability and translational efficiency of cellular mRNA targets (“targetome”) is increased in infected cells (**Figure 2**). The liver-specific miR-122 is an example of such a strategy adopted by HCV (Luna et al., 2015). MiR-122 binds two sites within the 5′-UTR of the HCV RNA genome and this binding moderately stimulates viral protein translation (Henke et al., 2008) while it protects the genome from XRN1-mediated degradation (Shimakami et al., 2012; Li et al., 2013; Sedano and Sarnow, 2014). In addition, miR-122 competes with cellular poly(rC)-binding protein 2 (PCBP2) binding to the HCV RNA genome and thereby promotes replication and packaging (Masaki et al., 2015). Similarly, pestiviruses hijack miR-17 and let-7 family members to promote their replication. Indeed, both let-7s and miR-17s directly interact with the 3′-UTR of the viral genome so as to stabilize the bovine viral diarrhea virus (BVDV) RNA and increase its translation (Scheel et al., 2016). Finally, miR-10a star strand (miR-10a-3p) directly targets CVB3 3D-coding sequence, thereby favoring its replication. Further *in vivo* investigations are required to elucidate the role of miR-10a-3p during CVB3 infection, in which a post-transcriptional regulation seems to be involved (Tong et al., 2013).

### Host miRNAs Can Act As Proviral Factors by Inhibiting Antiviral Host Factors

**FIGURE 3:**
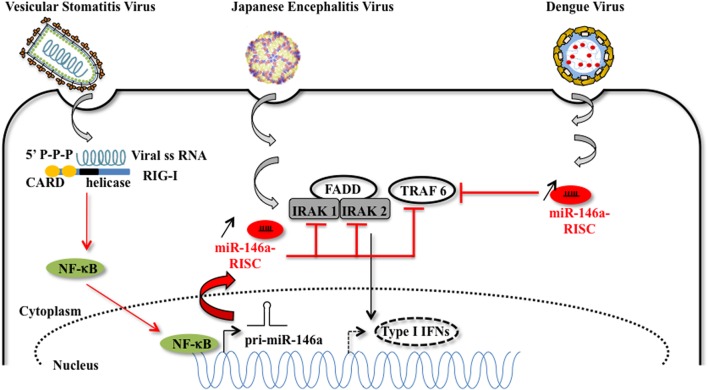
**Host miRNA-146a favors viral replication**. MiR-146a expression is increased upon vesicular stomatitis virus (VSV), Japanese encephalitis virus (JEV), and Dengue virus infection. In the case of VSV infection, miR-146a expression is increased in a RIG-I dependent manner. The RIG-I protein interacts with VSV RNA *via* its helicase domain, leading to the nuclear transcription of pri-miR-146a by NF-kB, and to an increasing amount of miR-146a. The proviral function of miR-146a is explained by the diminution of target mRNAs such as IRAK1, IRAK2, which are essential partners of the type I interferon response [image source for JEV adapted from Luca et al. (2012)].

**FIGURE 4:**
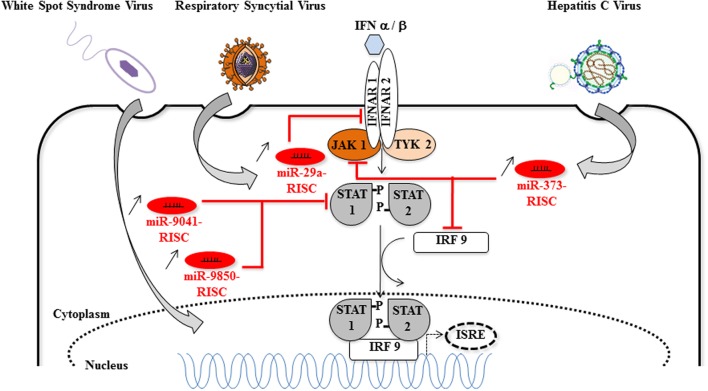
**Host miRNAs that favor viral replication *via* the inhibition of the Jak/STAT signaling pathway**. Cellular miRNAs (miR-9041, miR-9850, miR-29a, and miR-373) are upregulated during DNA [White spot syndrome virus (WSSV)] or RNA (respiratory syncytial virus, HCV) viruses infections. The proviral function of miR-9041, miR-9850, miR-29a, and miR-373 is explained by the diminution of target mRNAs coding for key elements of the JAK/STAT signaling pathway, such as STAT, IFNAR, JAK1, IRF9. Consequently, the formation of the STAT-IRF9 complex is impaired and the activity of the *ISRE* (interferon-stimulated response element) promoter is reduced (dotted arrows) [image source for HCV (Lindenbach and Rice, 2013)].

In DENV and VSV-infected monocytes/macrophages, and in JEV (strain JaOArS982) -infected human microglial brain cells (CHME3), the induction of miR-146a expression suppresses type I IFN production by targeting IL-1 receptor-associated kinase 1 (IRAK1), IRAK2, and tumor necrosis factor (TNF) receptor-associated factor 6 (TRAF6), thereby enhancing viral replication or allowing viral escape to cellular immune response (Hou et al., 2009; Wu et al., 2013; Zhang and Li, 2013; Sharma et al., 2015) (**Figure 3**). EV71 infection in RD cells also led to elevated levels of miR-146a. In this model, AP1 is the key transcription factor involved and miR-146a suppresses IRAK1 and TRAF6 expression leading to the inhibition of IFN production, and viral evasion to host immune attacks (Ho et al., 2014). White spot syndrome virus (WSSV) infection induces the up-regulation of miR-9041 and miR-9850, which in turn inhibit the JAK/STAT pathway, an inhibition resulting in the reduced expression of interferon-induced genes (Huang et al., 2016). HCV-dependent upregulation of miR-373 in hepatocytes is another example of a viral-induced inhibition of the JAK/STAT pathway, through targeting JAK1 and IFN-regulating factor 9 (IRF9) mRNAs (Mukherjee et al., 2015). Finally, type I interferon production is downregulated by non-structural proteins 1 and 2 (NS1 and NS2) encoded by RSV genome through the induction of miR-29a, which targets the IFN-alpha receptor (IFNAR1) 3′-UTR (Zhang et al., 2016) (**Figure 4**).

Altogether, host miRNAs influence the fate of viral infections, by inducing antiviral responses, by modulating cellular tropism or by playing a facilitating role in various phases of the viral life-cycles. However, because cellular miRNAs control multiple processes, their hijacking by viruses leads to the derepression of various cellular mRNAs, causing aberrant host protein expression. The most likely hypothesis is that viral adaptation was the result of host miRNA–mRNA interaction alterations, making the host cell environment favorable to viral persistence/chronicity (Li et al., 2014).

## Viral miRNAs (v-miRNAs)

Besides host miRNAs and the various roles they play in the virus–host interaction and resulting infection, certain viruses have been shown to generate their own miRNAs, which play a role in the viral life-cycles and may induce metabolic perturbations in the infected cells (**Table 1**).

**Table 1:** Viruses, v-miRNAs and their functions.

Viruses	v-miRNAs	Functions	Target RNA	Reference
**DNA viruses**		
γ-Herpesvirus saimiri (HVS)	miR-HSURs	Host cell cycle regulators repression	*P300 transcriptional activator, BiP, WEE1*	Guo et al., 2015
Murine Gammaherpesvirus 68 (MHV68)	mghv-miRNAs	Transition to latency in memory B cell and pathogenesis	Und.	Feldman et al., 2014
Ovine herpesvirus-2 (OvHV-2)	OvHV-2-miR-5	Latency	ORF50	Riaz et al., 2014
Herpes simplex virus 1 (HSV-1)	miR-H2-3p	Latency	ICP0	Umbach et al., 2008
Epstein–Barr virus (EBV)	BARTs	Associated-cancer development	LMP-1	Lo et al., 2007
		Latency	BZLF1, BRLF1, MAPKK2	Jung et al., 2014
Human cytomegalovirus (HCMV)	miR-UL112-1	Latency	IE1	Grassmann and Jeang, 2008
	miR-US4-1, miR-US5-1, miR-US5-2, miR-UL112-1	Antiviral immune response evasion	*ERAP 1, IL6, multiple endocytic pathway components*	Kim et al., 2011; Hook et al., 2014
Kaposi’s sarcoma-associated herpesvirus (KSHV)	miR-K-1	Latency	*IκBα*	Lei et al., 2010
	miR-K12-5, miR-K12-9, miR-K12-10a, miR-K12-11	Antiviral immune response evasion	*TWEAKR, IRAK1, MYD88, AID*	Abend et al., 2010, 2012; Bekerman et al., 2013
**Cytoplasmic RNA viruses**
West nile virus Kunjin (WNV_kun_)	Kun-miR-1	Viral replication enhancement	*GATA4*	Hussain et al., 2012
**Retroviruses**
Human immunodeficiency virus 1 (HIV-1)	HIV1-miR-H1	Cellular apoptosis induction	*AATF*	Kaul et al., 2009
		Viral protein R (Vpr) stabilization	*miR-149*	

*Host target genes are in italic, viral target genes are underlined, Und., undetermined*.

### v-miRNA Biogenesis

#### DNA Virus v-miRNAs

Since the discovery of the expression of miRNA by DNA viruses (Pfeffer et al., 2004), many research investigations have been conducted to identify and unravel the roles of these v-miRNAs. The functions described so far encompass regulation of viral persistence, proliferation and long-term survival of host cell and host immune evasion (Tycowski et al., 2015). Like eukaryotic miRNAs, v-miRNAs are generally processed by DROSHA and Dicer. In some cases, such as adenoviruses, pre-v-miRNAs can be directly transcribed and processed by Dicer, skipping the DROSHA step (Aparicio et al., 2006; Tycowski et al., 2015).

In HVS infection, which causes T-cell leukemias and lymphomas in new world primates, three pre-v-miRNAs are encoded immediately downstream of three Herpesvirus saimiri U snRNAs (HSURs 2, 4, and 5), forming chimeric HSV pri-snRNA/v-miRNAs. These chimeric transcripts are then processed by the Integrator host complex into pre-miRNAs from the 3′-end and Herpesvirus saimiri U snRNAs (HSURs) from the 5′-end extremities (Cazalla et al., 2011). These v-miRNAs, which do not bind to the HVS genome, repress many host mRNAs, preferentially those encoding cell cycle regulators (Guo et al., 2015).

Another class of DROSHA-independent miRNAs includes the Murine Gammaherpesvirus 68 (MHV68) v-miRNAs which are essential for transition to latency in the key virus reservoir of memory B-cells and for pathogenesis (Feldman et al., 2014). These v-miRNAs are transcribed as chimeric pri-RNAs consisting of tRNAs linked to pre-microRNA hairpins. Host tRNaseZ cleavage removes the tRNA and frees the pre-microRNA for Dicer processing (Bogerd et al., 2010; Diebel et al., 2010).

#### Cytoplasmic RNA Virus v-miRNAs

Although this is debated, it appears that some cytoplasmic RNA viruses could generate v-miRNAs (Swaminathan et al., 2013). The WNV Kunjin strain contains a sfRNAs in the 3′-UTR of its genomic RNA with several stem–loops that serve as Dicer substrates for the generation of v-miRNAs called KUN-miR-1. These v-miRNAs enhance viral replication (Hussain et al., 2012).

The discovery that cytoplasmic RNA viruses could be engineered to produce functional miRNAs emphasizes the existence of cytoplasmic, non-canonical, miRNA biogenesis pathways. For instance, infection of BHK cells by cytoplasmic recombinant Sindbis viruses (rSINVs) allows the expression of virus-derived cytoplasmic pri-miRNAs (c-pri-miRNAs). The viral-induced translocation of DROSHA into the cytoplasm initiates the processing of these c-pri-miRNAs and the biogenesis of miRNAs (Shapiro et al., 2012).

#### Retrovirus v-miRNAs

Retroviruses constitute a class of positive-sense ssRNA viruses, ubiquitous in nature, causing cancers and immunodeficiency syndromes, which are reverse transcribed to dsDNAs that integrates the host genome (Zheng et al., 2012). The capacity of Human immunodeficiency virus 1 (HIV-1) to encode v-miRNAs has been extensively studied both *in silico* and *in vivo* (Bennasser et al., 2004). HIV1-miR-H1 is generated from a pre-miRNA sequence within the 3′-end of the viral genome. In human mononuclear cells, this v-miRNA has been reported to selectively target the apoptosis antagonizing transcription factor (AATF) transcript, thereby suppressing the expression of c-myc, Par-4, Bcl-2 and the RISC protein Dicer. This v-miRNA also downregulates the host miR-149, which targets the viral accessory protein Vpr (Kaul et al., 2009). A 50 nt HIV-1 TAR motif located within the 5′ end of the viral genome is also a source of several v-miRNAs (Narayanan et al., 2011). Other retroviruses than HIV generate v-miRNAs using either canonical or non-canonical miRNA biogenesis pathways. They include the avian leukosis virus (Lakatos et al., 2006; Yao et al., 2014), the bovine leukemia virus (BLV), the bovine foamy virus (BFV) (Kincaid et al., 2012; Burke et al., 2014; Whisnant et al., 2014), and the simian foamy virus (SFV) (Tycowski et al., 2015).

### v-miRNAs Functions

#### Regulation of Viral Life-Cycles

Several v-miRNAs are generated from the antisense strand of viral protein-coding genes, showing a perfect match to their target mRNAs. In general, these antisense v-miRNAs are involved in the lytic or latency phase transition (Grassmann and Jeang, 2008). For instance, EBV expresses the BamHI-A antisense transcripts (BARTs) that produces v-miRNAs. The EBV BARTs produce two clusters of miRNAs (12 and 15 v-miRNAs in Clusters 1 and 2, respectively). It was reported that BART cluster 1 miRNAs target the viral LMP-1 3′-UTR, a prime candidate for driving nasopharyngeal carcinoma (NPC) development. Negative regulation of LMP1 expression may thus favor EBV-associated cancer development (Lo et al., 2007). Moreover, BART20-derived v-miRNA promotes the latency phase by targeting two EBV immediate-early genes, including BZLF1 and BRLF1 (Jung et al., 2014).

Human cytomegalovirus lytic replication is regulated by miR-UL112-1 that targets the IE1 (immediate early viral protein) 3′-UTR, thereby sustaining the latency phase (Grassmann and Jeang, 2008). Likewise, the ovine herpesvirus v-miRNA, OvHV-2-miR-5, targets ORF50 mRNA and maintains latency (Riaz et al., 2014). A v-miRNA-dependent strategy is also employed by HSV-1, which encodes miR-H2-3p that directly targets latency-associated transcripts, including the viral immediate early gene transactivator (Umbach et al., 2008).

#### Host mRNA Regulation

Viral-miRNAs can also regulate the expression of host mRNAs. v-miRNAs from KSHV target different host mRNAs, including the IκBα mRNA, activating NF-κB signaling and preventing viral lytic replication (Lei et al., 2010). Similarly, the EBV specific BART18-derived v-miRNA targets the MAPK kinase kinase 2 mRNA, thereby preventing the initiation of lytic viral replication (Qiu and Thorley-Lawson, 2014). Other EBV- or KSHV-expressed v-miRNAs reduce the expression of pro-apoptotic proteins or inhibit cell cycle progression (Gottwein and Cullen, 2010). Notably, v-miRNAs from some gamma-herpesviruses, such as KSHV and Marek’s disease virus type 1 (MDV-1), or retroviruses, such as SFV, share the same seed-sequence of miR-155 and regulate the miR-155 targetome (Guo and Steitz, 2014).

Several v-miRNAs enhance viral survival by targeting genes involved in the antiviral immune response. For example, the KSHV-expressed miR-K12-10a directly reduces the expression of TWEAKR (TNF-like weak inducer of apoptosis receptor) (Abend et al., 2010), whereas miR-K12-9 and miR-K12-5 target the TLR/Interleukin-1R signaling cascade at two distinct points (IRAK1 and MYD88), thereby reducing inflammatory cytokine mRNAs expression (Abend et al., 2012). The KSHV-expressed miR-K12-11 and miR-K12-5 prevent the recognition of infected cells by natural killer (NK) cells by directly targeting the activation-induced cytidine deaminase (AID) coding gene (Bekerman et al., 2013). Similarly, HCMV-expressed v-miRNAs favors the evasion from the NK antiviral response (Nachmani et al., 2009). The HCMV v-miR-US4-1 alters the MHC class I presentation pathway by targeting endoplasmic reticulum-resident aminopeptidases, such as ERAP1, a “molecular ruler” for antigenic peptide production in T-lymphocytes (Kim et al., 2011). Moreover, HCMV-expressed miR-UL112-1, miR-US5-1, and miR-US5-2 control mRNAs encoding IL6 and TNF-α, which are involved in the secretory pathways, thereby altering the secretion of host cytokines and ultimately blocking the antiviral response (Hook et al., 2014).

These observations indicate that viruses, including DNA viruses, cytoplasmic RNA viruses and retroviruses, produce v-miRNAs through or independently of the DROSHA-dependent maturation pathways. These v-miRNAs generally play a proviral role by enhancing the viral life-cycles, impairing the expression of genes involved in antiviral responses or regulating cell metabolism (**Table 1**). Thus, specific v-miRNAs could represent an interesting target for antiviral interventions.

## Therapeutic Manipulation of miRNAs for Antiviral Therapy

Recent advances in the field of antiviral therapy consist of specifically inhibiting a viral component in order to block the viral life-cycle, and thereby inhibiting viral production. The roles played by several host miRNAs and by v-miRNAs pave the way to specific antagomirs approaches. Such an approach has been applied to HCV in several *in vitro* and *in vivo* proof-of-concept studies. A specific inhibitor (antagomir) of miR-122 [miravirsen, Santaris Pharma, Hørsholm, Denmark] entered into human clinical trials (Janssen et al., 2013). In a phase II trial, 5 weekly miravirsen injections induced a dose-dependent reduction of HCV RNA levels ranging from 1.2 to 3.0 Log international units (IUs)/mL, which was maintained after treatment cessation. Five out of 27 patients still had undetectable HCV RNA 14 weeks after the end of therapy. In a recent trial (van der Ree et al., 2017), RG-101, a hepatocyte targeted *N*-acetylgalactosamine conjugated oligonucleotide that antagonizes miR-122, inhibited viral replication by up to 5 Log IU/mL after one single injection of the antagomir, and 3 out of 32 patients were even cured of the infection. These very promising results suggest that antagomir-based approaches are susceptible to profoundly and sustainably inhibit viral replication.

Currently, a major barrier to the application of miRNA/siRNA-based therapies is the non-toxic delivery to infected sites (Tahamtan et al., 2016). Delivery of sufficient amounts of miRNA/anti-miRNA molecules is indeed challenging. Host miRNAs are key regulators of gene expression, and their long-term manipulation may predispose one to cellular abnormalities, impaired immunity, or even cell transformation. Therefore, side-effects correlated with long-term suppression/overexpression of host miRNAs may limit the clinical use of such strategies. Targeting v-miRNAs rather than host miRNAs could be a reasonable alternative in some cases. More preclinical and early clinical studies are now warranted.

## Conclusion and Perspectives

In summary, we are far from fully understanding the molecular mechanisms underlying the complex crosstalks between miRNA pathways and viral infections. Deciphering the pathways of v-miRNAs generation, for DNA or RNA viruses, remains challenging as well. However, knowledge is increasing on the diverse roles played by either host miRNAs or v-miRNAs during viral infections. It is now necessary to decipher virus/miRNA networks and unravel the detailed miRNA/v-miRNA mechanism(s) during viral infections and the antiviral response, in order to improve our antiviral *armamentarium* to cure or control a number of viral infections without current therapies.

## Author Contributions

All authors contributed to the planning, writing, and revision of the manuscript. All authors read and approved the manuscript.

## Conflict of Interest Statement

The authors declare that the research was conducted in the absence of any commercial or financial relationships that could be construed as a potential conflict of interest.
